# Long non-coding RNA crnde promotes deep vein thrombosis by sequestering miR-181a-5p away from thrombogenic Pcyox1l

**DOI:** 10.1186/s12959-023-00480-9

**Published:** 2023-04-19

**Authors:** Xin He, Yu Liu, Yaozhen Li, Kemin Wu

**Affiliations:** 1 Department of Anesthesiology, Xiangya Hospital, Central South University, Changsha, 410008 Hunan Province China; 2 Department of General and Vascular Surgery, Xiangya Hospital, Central South University & National Clinical Research Center for Geriatric Disorders, Changsha, 410008 Hunan Province China

**Keywords:** High-throughput transcriptome sequencing, Deep vein thrombosis, ceRNA regulatory network, Long non-coding RNA crnde, microRNA-181a-5p, Pcyox1l, Vascular inflammatory injury

## Abstract

**Background:**

Deep vein thrombosis (DVT) is an interplay of genetic and acquired risk factors, where functional interactions in lncRNA-miRNA-mRNA ceRNA networks contribute to disease pathogenesis. Based on the high-throughput transcriptome sequencing prediction, we have assessed the contribution of lncRNA Crnde/miR-181a-5p/Pcyox1l axis to thrombus formation.

**Methods:**

DVT was modeled in mice by inferior vena cava stenosis, and inferior vena cava tissues were harvested for high-throughput transcriptome sequencing to screen differentially expressed lncRNAs and mRNAs. The key miRNA binding to Crnde and Pcyox1l was obtained through searching the RNAInter and mirWalk databases. The binding affinity between Crnde, miR-181a-5p, and Pcyox1l was examined by FISH, dual luciferase reporter gene, RNA pull-down, and RIP assays. Functional experiments were conducted in DVT mouse models to assess thrombus formation and inflammatory injury in inferior vena cava.

**Results:**

It was noted that Crnde and Pcyox1l were upregulated in the blood of DVT mice. Crnde competitively bound to miR-181a-5p and inhibited miR-181a-5p expression, and Pcyox1l was the downstream target gene of miR-181a-5p. Silencing of Crnde or restoration of miR-181a-5p reduced inflammatory injury in the inferior vena cava, thus curtailing thrombus formation in mice. Ectopic expression of Pcyox1l counterweighed the inhibitory effect of Crnde silencing.

**Conclusions:**

Therefore, Crnde sequesters miR-181a-5p to release Pcyox1l expression *via* ceRNA mechanism, thus aggravating thrombus formation in DVT.

**Supplementary Information:**

The online version contains supplementary material available at 10.1186/s12959-023-00480-9.

## Introduction

Deep vein thrombosis (DVT), attributed to changes in venous homeostasis, presents a common reason of cardiovascular mortality [1]. DVT usually occurs in the lower extremities but can also occur in other sites [2]. In spite of anticoagulant therapy, many DVT patients may suffer from post-thrombotic syndrome [3]. Interaction of platelets with monocytes and neutrophils can induce DVT [4]. Moreover, DVT is related to the predominance of inflammatory cells [5]. It is clear that inflammation has a key role in the pathophysiology of DVT [6, 7]. Therefore, seeking novel targets for managing inflammation in DVT is required.

It is noteworthy that the transcriptome high-throughput sequencing performed in the current study predicted the long non-coding RNA (lncRNA) colorectal neoplasia differentially expressed (Crnde) as a pivotal gene in DVT. Crnde, a type of 1910-nt lncRNA encoded on human chromosome 16, is implicated in multiple cancers [8]. Upregulation of Crnde was found in injured rat carotid artery as well as vascular smooth muscle cells induced by platelet-derived growth factor-BB [9]. Crnde as a potential biomarker induced inflammation in alcoholic liver disease [10].

Prenylcysteine oxidase 1 (Pcyox1) is a type of enzyme responsible for prenylated protein degradation and release of hydrogen peroxide, cysteine and isoprenoid aldehyde [11]. Pcyox1 can be expressed in vascular and blood cells, and the lack of Pcyox1 brought about platelet hypo-reactivity as well as impaired arterial thrombosis [12].

Of note, our bioinformatics analysis screened microRNA (miR)-181a-5p as the differentially expressed one in DVT which could bind to both Crnde and Pcyox1l (the paralogous gene of Pcyox1. Strikingly, a previous study demonstrated that Crnde sponged miR-181a-5p, thereby leading to aggravation of inflammation underlying sepsis [13]. Moreover, upregulation of miR-181 could diminish the luciferase activity in cells with FXI-3’UTR and might serve as a novel therapeutic target for prevention of thrombosis [14]. Interestingly, it was previously unfolded that miR-181a-5p in cooperation with miR-181a-3p could repress vascular inflammation as well as atherosclerosis [15]. Moreover, miR-181a-5p alleviated inflammatory response in pulmonary arterial hypertension induced by monocrotaline, which was achieved by targeting endocan [16]. In view of the aforementioned reports, we thus proposed a hypothesis in the current study that lncRNA Crnde might affect vascular inflammation injury in DVT by regulating the miR-181a-5p/Pcyox1l axis.

## Materials and methods

### Ethical approval

This study was performed under the approval of the Ethics Committee of Xiangya Hospital, Central South University. All animal experiments were conducted strictly following the *Guide for the Care and Use of Laboratory Animals*.

### Establishment of mouse DVT model

Fifty-four BALB/C mice aged 4–6 weeks old (18–22 g) (Hunan SJA Laboratory Animal Co., Ltd., Changsha, China) were housed for 2 weeks in a specific-pathogen-free environment at a constant room temperature of 20–25℃ with constant 60–65% humidity. Mice were acclimatized to the pre-experimental environment with free access to food and water under 12-h dark/light cycles.

The mouse DVT model was constructed by inferior vena cava stenosis under sevoflurane inhalation anesthesia. Mice were fixed in a supine position on the operating table and their abdomen was shaved and sterilized with a 0.5% povidone-iodine solution. The medial skin of the thigh was cut longitudinally, and the femoral veins were exposed at 2 cm depth, with a mosquito clamp holding three different veins. The mouse model was established after the suture incision. Sham-operated mice underwent a longitudinal suture incision of the medial skin of the thigh [17, 18]. Mice were given normal feeding after they regained consciousness.

### Transcriptome high-throughput sequencing and data processing

Inferior vena cava tissue samples from DVT mice (lesion area of the thrombus) and control mice (n = 3) were collected to a vacuuming tube containing 3.8% sodium citrate, and after blood sampling, full transcriptome sequencing of six samples was completed on the high-throughput sequencing platform Illumina. Trimmomatic tool was used for quality pretreatment of raw data: (1) removal of the adaptor; (2) removal of low-quality reads; (3) removal of low-quality bases from the 3′ and 5′ ends; (4) counting original sequencing amount, effective sequencing amount, Q30 and GC content, and summarizing the number of reads in the whole quality control process. Sequence alignment was performed between the filtered high-quality reads and the mouse genome using hisat2 software.

### Differential gene expression analysis and enrichment analysis

Differential expression analysis of lncRNAs and mRNAs was performed using the R language “edgeR” package based on the read counts of the lncRNAs and mRNAs, with |log_2_FC| > 1 and *p* < 0.05 set as the criteria. Subsequently, six differentially expressed lncRNAs and six differentially expressed mRNAs were selected for RT-qPCR to verify whether the transcriptome data were reliable.

The volcano plots were then drawn using the “ggplot2” package in R language and the differential gene expression heatmaps using the R software “heatmap” package. GO and KEGG enrichment analyses of mRNAs were performed by the R language “clusterProfiler” package.

### Lentiviral vectors

Lentiviral vectors expressing short hairpin RNA (sh)-negative control (NC), sh-Crnde, agomir NC, miR-181a-5p agomir, sh-NC + overexpression (oe)-NC, sh-Crnde + oe-NC and sh-Crnde + oe-Pcyox1l were injected into the mice via the tail vein 1 day before model construction [19]. These lentiviruses (150 µL for each group) were all purchased from GenePharma (Shanghai, China). The nucleotide sequence of Crnde was obtained by NCBI, and the Crnde interference sequences were designed by the ThermoFisher database (Table S1).

Mouse DVT model was constructed five days after lentivirus injection. Mice were sham-operated (n = 6), or modeled as DVT mice without treatment (n = 6), or treated with sh-NC (n = 6), sh-Crnde (n = 6), agomir NC (n = 6), miR-181a-5p agomir (n = 6), sh-NC + oe-NC (n = 6), sh-Crnde + oe-NC (n = 6) or sh-Crnde + oe-Pcyox1l (n = 6).

### Measurement of weight and length of thrombus

Seven days after molding, mice were anesthetized with intraperitoneal injection of 3% pentobarbital sodium and were euthanized by cervical dislocation. Afterwards, the mice were fixed to the operating table in a supine position. The skin of the medial thigh was cut vertically, the femoral vein was exposed to a 2 cm incision, and the femoral vein was cut. The thrombus was removed from the inferior vena cava to observe the thrombus characteristics and ablation. The weight (mg) and the length of the thrombus (mm) were measured.

### ELISA for serum levels of inflammatory factors

Mouse serum levels of IL-1β, IL-6, and IL-8 were determined using the ELISA kit for IL-1β (ab100704, Abcam, Cambridge, UK), IL-6 (ab100712, Abcam), and IL-8 (SEKM-0046, Solarbio, Beijing, China), referring to the kit instructions [20]. Absorbance was obtained at 450 nm using a microplate reader (800TS, BioTek, Winooski, VT) and analyzed using the Origin 9.5 software.

### HE staining

Inferior vena cava tissues from mice were fixed with 10% neutral formaldehyde for more than 24 h, embedded in paraffin, and sliced into serial Sect. (5 μm). Hematoxylin (H8070-5 g, Solarbio) was used to stain the sections for 4 min, followed by eosin solution (PT001, Shanghai Bogoo Biological Technology Co., Ltd., Shanghai, China) staining for 2 min [21]. The morphology was observed under an optical microscope (Olympus BX51, Olympus, Tokyo, Japan).

### Western blot and immunohistochemistry

Protein in the inferior vena cava tissue was extracted with an extraction kit (EX2410, Solarbio) and determined using the BCA Protein Assay kit (Pierce; Thermo Fisher Scientific, Rockford, IL). Proteins (20 µg per lane) were subjected to 10–12% SDS-PAGE and transferred to a polyvinylidene difluoride membrane. The membrane was then blocked with 5% BSA for 2 h, and incubated overnight at 4℃ with the primary anti-Pcyox1l antibody (0.5 mg/mL, PA5-57955, 0.4 µg/mL, Thermo Fisher Scientific). After washing, the membrane was incubated with a goat anti-rabbit horseradish peroxidase-conjugated secondary antibody (2 mg/mL, ab6721, 1 : 2000, Abcam) for 2 h at room temperature and developed using an enhanced chemiluminescence system (Pierce; Thermo Fisher Scientific). Anti-GAPDH antibody (2 mg/mL, ab8245, 1 : 500-1 : 10,000, Abcam) was used as an internal reference.

For antigen retrieval, the sections of mouse thrombus and inferior vena cava tissues were treated with 0.3% H_2_O_2_ methanol treatment for 10 min, washed with distilled water, immersed into 0.01 M citrate buffer solution (PH6.0), and radiated with microwave in a microwave oven for 10 min. After the retrieval solution was cooled down to room temperature, the sections were dripped with normal goat serum and incubated at 4℃ overnight with the following antibodies: anti-Pcyox1l antibody (0.5 mg/mL, PA5-57955, 1 : 500-1 : 1000, Thermo Fisher Scientific), F4/80 monoclonal antibody (0.5 mg/mL, ab300421, 1 : 5000, Abcam), myeloperoxidase polyclonal antibody (0.5 mg/mL, ab208670, 1 : 1000, Abcam). Next, the sections were further incubated with secondary antibody goat anti-rabbit IgG (2 mg/mL, ab6721, 1 : 2000, Abcam) at 37℃ for 20 min. Horseradish peroxidase-labeled streptavidin was used to incubate the sections at 37℃ for 20 min, followed by DAB (ST033, Whiga, Guangzhou, China) color development. Hematoxylin (PT001, Shanghai Bogoo Biological Technology Co., Ltd.) was used for counterstaining the sections, and Images were observed and photographed under a microscope.

### RT-qPCR

Total RNA was extracted from the inferior vena cava tissue [22]. For RNA, First Strand cDNA Synthesis Kit (K1622, Fermentas, Hanover, MD) was used for reverse transcription of 1 µg of total RNA into cDNA. For miRNA, PolyA Tailing Kit (B532451, Sangon, Shanghai, China) was used for reverse transcription into cDNA. The synthesized cDNA was detected by RT-qPCR with the Fast SYBR Green PCR kit (Applied Biosystems) and the ABI PRISM 7500 RT-PCR system (Applied Biosystems), with three replicates set for each well. The 2^−ΔΔCt^ method was utilized to analyze the relative gene expression as normalized to GAPDH and U6. The sequences of the primers used for the experiments are shown in Table S2. The materials used in the above test steps were purchased from Servicebio, Wuhan, China.

### Isolation, culture and identification of mouse primary vascular endothelial cells

We isolated and purified mouse primary vascular endothelial cells as previously described [23]. In brief, a 6-well plate was put on ice, and 1 mL of matrix was applied on one well of the plate without introducing any bubbles. The plate was incubated in an incubator at 37℃ for 20 min to solidify the substrate. The tissue was implanted into the coagulation matrix using sterile microanatomy forceps. The lumen was placed on the matrix downward without touching the endothelium, and 3–4 tissue blocks were placed on the matrix close to each other. Next, cells were cultured with 200 µL of endothelial cell growth medium (C0065C, Gibco, Carlsbad, CA) at 37℃ with 5% CO_2_ for 4–6 h, followed by addition of medium to reach 1 mL. On the 4th day, the medium and tissue blocks were removed, and 2 mL of new endothelial cell growth medium was added for allowing the endothelial cells to further proliferate on the matrix for 2–3 days. Subsequently, the primary endothelial cells were transferred to a T12.5 flask covered with 0.1% gelatin (#07903, STEMCELL Technologies, Shanghai, China), further cultured with 4 mL of endothelial cell growth medium and stably passaged 3 times.

Identification of primary endothelial cells: a 6-well plate was paved with 0.1% gelatin, and then 3 × 10^5^ cells were seeded in each well. After 24 h of culture at 37℃ with 5% CO_2_, the cell morphology and adherence were observed under a light microscope. Cells were taken out from the culture plate and fixed with 1 mL of 10% formaldehyde at room temperature for 30 min. Next, 1 mL of PBS was used to suspend the cells, followed by incubation with anti-CD31 (0.5 mg/mL, ab222783, 1:100, Abcam) and anti-VE-Cadherin (0.2 mg/mL, ab205336, 1:1000, Abcam) on ice in darkness for 1 h. The cells were incubated with the secondary antibody against IgG (1.7 mg/mL, ab172730, 1:100, Abcam) on ice in darkness for 1 h and then with 1 ug/mL DAPI in dark for 10 min. Finally, the fluorescence intensity and distribution were observed under a fluorescence microscope to identify the purity of endothelial cells. Figure S1A shows that the primary endothelial cells had good spindle-shaped and pebble-shaped morphology. Figure S1B shows that about 95% of primary endothelial cells expressed endothelial marker proteins CD31 and VE-Cadherin, suggesting successful isolation of primary endothelial cells with high purity.

### FISH assay

The FISH technique was used to determine the localization of Crnde with miR-181a-5p in the cells. Crnde was labeled by a Cy5 probe and fam probe was used to label miR-181a-5p. The probes were designed and synthesized by GenePharma and a FISH kit (GenePharma) was utilized to detect the signal of the probes according to the manufacturer’s instructions. Images were taken with a Lei TCS SP8 laser scanning confocal microscope (Leica Microsystems, Mannheim, Germany). The cell experiments were independently repeated three times.

### Dual luciferase reporter gene assay

Cells were transfected with the luciferase reporter plasmid containing Pcyox1l with the wild-type or a mutated version of the binding site and mimic-NC or miR-181a-5p mimic with Lipofectamine 2000 (Invitrogen). After 48 h of transfection, the cells were collected to detect luciferase activity using the Dual-Luciferase reporter assay system (Promega) as normalized to Renilla luciferase activity [24]. The cell experiments were independently repeated three times.

### RNA pull-down assay

A Biotin-labeled Crnde probe (Crnde probe) and a NC probe were purchased from Sangon. The Crnde or NC probe was incubated with magnetic beads at 4℃. An equal number of cells were seeded in two 10-cm culture dishes, respectively. After 24 h, the cells on the two culture dishes were collected for lysis, and 50 µL of cell lysates were frozen at -80℃ as Input. Cell lysates were then incubated for 1 h at room temperature with a magnetic bead mixture. After purification, the enrichment of RNA was determined by RT-qPCR. Data of the Input of the two groups of samples were standardized, and then the relative expression of Crnde and miR-181a-5p in the pull-down NC probe and Crnde probe samples was calculated based on the Ct value of each input sample. The cell experiments were independently repeated three times.

### RNA immunoprecipitation (RIP) assay

Magna RIP RNA-Binding Protein Immunoprecipitation Kit (Millipore, Burlington, MA) for RIP assay. Cells were cultured with NP-40 RIP lysate buffer containing DTT (1 mM), PMSF (1 mM), RNase inhibitor (200 U/mL) and 1% protease inhibitor. RIP buffer with magnetic beads conjugated with AGO2 antibody (0.5 mg/mL, ab186733, 1:30, Abcam) to the whole cell lysate (200 µL), with IgG antibody (2 mg/mL, ab205718, 1:50, Abcam) serving as the NC. The beads were rinsed with pre-cooled NT2 buffer and incubated with protease K (10 mg/mL) for 30 min to prevent non-specific binding. The immunoprecipitated RNA was purified and the levels of miR-181a-5p and Pcyox1l mRNA were detected by RT-qPCR. The cell experiments were independently repeated three times.

### Statistical analysis

All data were processed using SPSS 21.0 statistical software (IBM Corp. Armonk, NY). Measurement data were represented in the form of mean ± standard deviation. Data comparisons between two groups were conducted by unpaired *t* test, and those among multiple groups by one-way ANOVA with Tukey’s post hoc tests. *p* < 0.05 indicated significant differences.

## Results

### High-throughput transcriptome sequencing analysis identified the involvement of Crnde in DVT

**Fig. 1:**
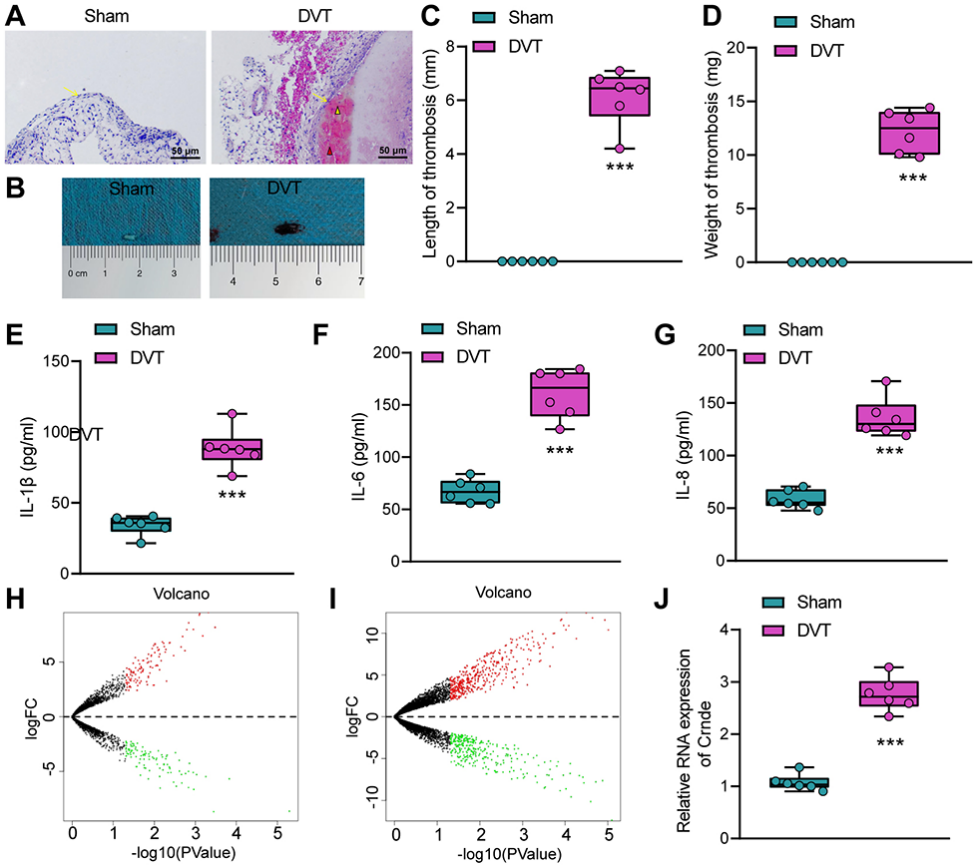
High-throughput transcriptome sequencing analysis to screen key genes involved in DVT. A, DVT in the sham-operated mice and DVT mice observed by HE staining. White triangles indicate the platelet bundles, red triangles indicate clustered red blood cells, and yellow arrows indicate the blood vessel wall. B, Representative image of thrombosis in the inferior vena cava of the sham-operated mice and DVT mice. C & D, Measurement results of the length (C) and weight (D) of thrombus in the sham-operated mice and DVT mice (n = 6). E-G, The levels of inflammatory factors IL-1β (E), IL-6 (F) and IL-8 (G) in mouse femoral venous blood of the sham-operated mice and DVT mice measured by ELISA (n = 6). H & I, Volcano plots of differentially expressed lncRNAs (H) and mRNAs (I) analyzed by high-throughput sequencing (Black dots represent genes not differentially expressed, red dots represent the upregulated genes, and green dots represent downregulated genes. n = 3). J, The Crnde expression in DVT mice measured by RT-qPCR (n = 6). *** *p* < 0.001 vs. the sham group

We established a mouse DVT model. HE staining results (Fig. 1A) showed that the sham-operated mice had intact wall of the inferior vena cava, without inflammatory cell infiltration, accumulation of platelets and red blood cells, or formation of thrombosis; however, in DVT mice, thrombosis formed in the inferior vena cava, accompanied by inflammatory cell infiltration around the thrombus and around the venous wall. In addition, the thrombus length (mm) and thrombus weight (mg) measurements (Fig. 1B-D) showed that mean thrombus length was 5.97 mm and mean thrombus weight was 13.32 mg. The ELISA results found that the levels of IL-1β, IL-6, and IL-8 were increased significantly in the femoral venous blood of the DVT mice compared with those in the sham-operated mice (Fig. 1E-G). The above results indicated the successful establishment of the mouse DVT model.

Next, we collected inferior vena cava tissue samples from DVT mice and control mice for high-throughput transcriptome sequencing. Differential analysis selected 211 differentially expressed lncRNAs (Fig. 1H) and 714 differentially expressed mRNAs (Fig. 1I). lncRNA Crnde was ranked high in the transcriptome data in terms of differential expression, and the expression of lncRNA Crnde in the inferior vena cava of DVT mice was notably higher than that in the sham-operated mice (Fig. 1J). It has been previously reported that lncRNA Crnde participates in many biological processes, such as cell proliferation, differentiation, migration and apoptosis [9, 25], and promotes the inflammation [10, 26, 27]. Therefore, we selected Crnde as the target gene for subsequent experiments.

### Silencing of Crnde attenuated vascular inflammatory injury, thereby curtailing thrombus formation

**Fig. 2:**
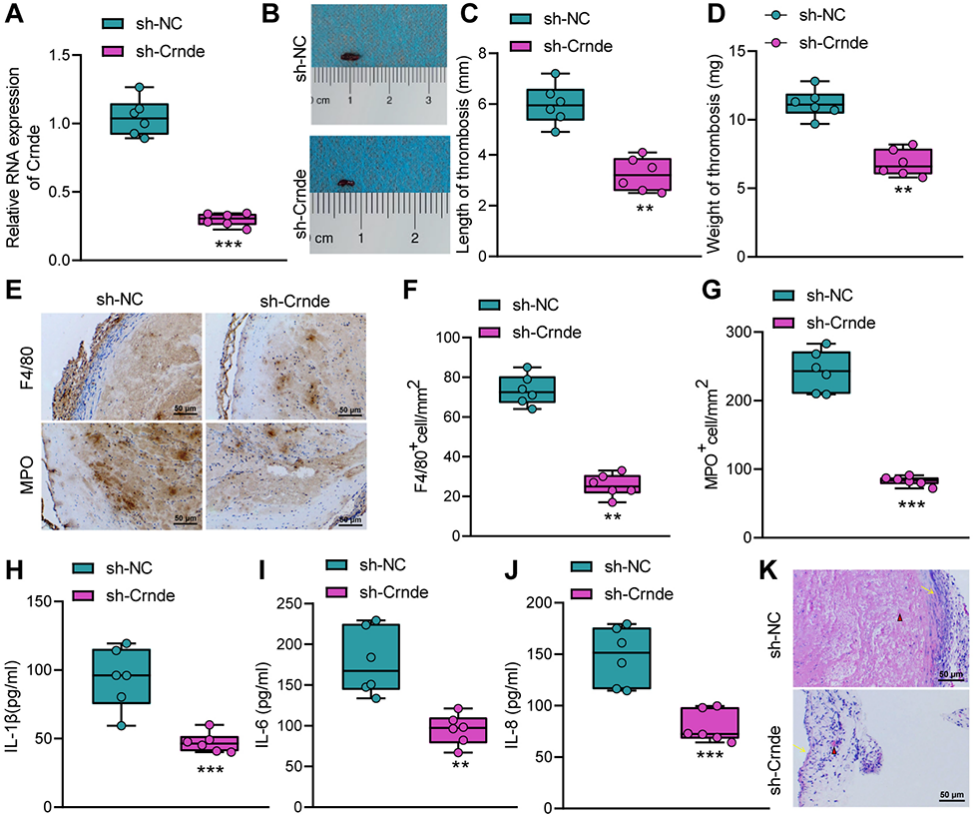
Silencing of Crnde attenuates vascular inflammatory injury, thereby curtailing DVT. A, RT-qPCR detection of Crnde expression in the inferior vena cava tissues of the sh-Crnde-treated DVT mice. B, Representative image of thrombosis in inferior vena cava of the DVT mice in response to Crnde knockdown. C & D, Measurement results of length (C) and weight (D) of thrombus in the DVT mice in response to Crnde knockdown. E, Representative micrographs of thrombus (F4/80) and myeloperoxidase staining for the DVT mice in response to Crnde knockdown. F, Quantification of F4/80 positive cells per unit area in the inferior vena cava tissues of the DVT mice in response to Crnde knockdown. G, Quantification of myeloperoxidase staining-positive cells per unit area in the inferior vena cava tissues of the DVT mice in response to Crnde knockdown. H-J, The levels of inflammatory factors IL-1β (H), IL-1, IL-6 (I) and IL-8 (J) in the femoral venous blood of the DVT mice in response to Crnde knockdown. K, The vascular inflammatory injury and platelet bundles in the DVT mice in response to Crnde knockdown. Red triangles indicate clustered red blood cells, and yellow arrows indicate the blood vessel wall. ** *p* < 0.01, *** *p* < 0.001 vs. the sh-NC group. n = 6

To elucidate the role of Crnde in DVT, we injected either lentiviral vectors expressing sh-NC or sh-Crnde in the DVT mice to construct a sh-Crnde mouse DVT model. RT-qPCR results revealed that Crnde gene knockdown had a significant effect in mice, and mice treated with sh-Crnde had reduced Crnde expression (Fig. 2A).

Furthermore, we also observed that DVT mice in response to Crnde knockdown had decreased length and weight of the thrombosis in inferior vena cava (Fig. 2B-D). Immunohistochemical staining displayed that knockdown of Crnde contributed to reduced inflammatory cells, especially macrophages and neutrophils in thrombosis (Fig. 2E-G). Based on the ELISA results, the levels of IL-1β, IL-6, and IL-8 were suppressed by knockdown of Crnde (Fig. 2H-J). The HE staining results (Fig. 2K) showed that the DVT mice in response to Crnde knockdown had superficial staining of the thrombosis, diminished platelet bundles, and decreased inflammatory cell infiltration. Collectively, silencing of Crnde curtailed thrombus formation by attenuating vascular inflammatory injury.

### Bioinformatics analysis identified the involvement of the Crnde/miR-181a-5p/Pcyox1l ceRNA network in DVT

**Fig. 3:**
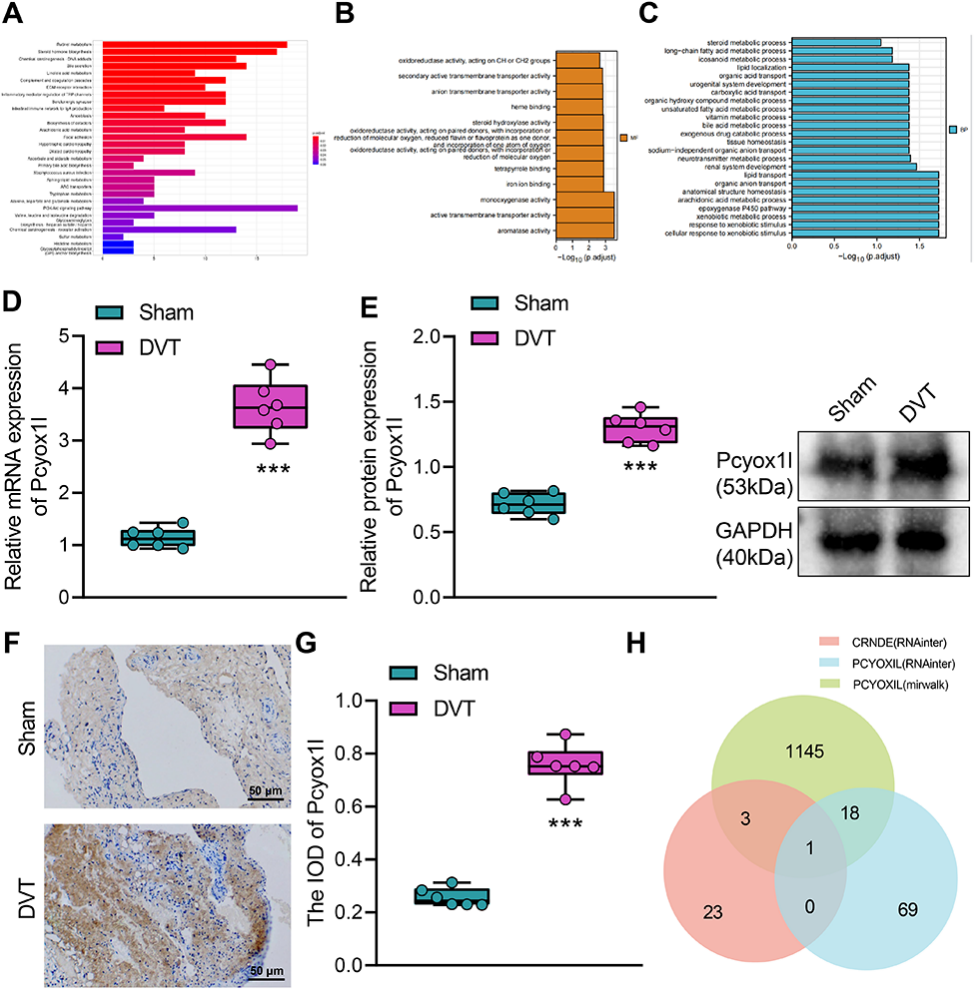
Bioinformatics analysis for and construction of the ceRNA regulatory network involved in DVT. A, KEGG enrichment analysis diagram of 714 differentially expressed mRNAs. B & C, Plots of molecular function (B) and biological process (C) for the GO enrichment analysis of the 714 differentially expressed mRNAs. D & E, The mRNA (D) and protein (E) expression of Pcyox1l in DVT mice measured by RT-qPCR and Western blot. F & G, Images (F) and quantitation (G) of positive rate of Pcyox1l protein expression in DVT mice determined by immunohistochemistry. H, Venn diagram showing the miRNAs predicted by RNAInter and mirWalk databases. *** *p* < 0.001 vs. the sham group. n = 6

Next, we performed a functional enrichment analysis on the 714 differentially expressed mRNAs. KEGG enrichment found that the 714 differentially expressed mRNAs were mainly enriched in pathways such as cell adhesion molecules, inflammatory mediator regulation, and complement and coagulation cascade (Fig. 3A), all of which were associated with platelet function [28]. In addition, GO enrichment analysis found that the 714 differentially expressed mRNAs were mainly involved in signal transduction, oxidoreductase activity and other life activities related to platelet function (Fig. 3B, C).

In addition, Pcyox1l ranked top in importance among the 714 differentially expressed mRNAs (logFC = 4.119, *p* = 0.001). Therefore, we chose Pcyox1l as the downstream target gene for subsequent studies. Pcyox1l is the paralogous gene of Pcyox1 [11] which is involved in the degradation of prenylated protein enzymes, expressed in different tissues like blood vessels and blood cells, and involved in the regulation of peptidase activity, platelet degranulation, signal transduction, stress response, stimulation response, inflammation and injury related life regulation activities [11]. We measured the Pcyox1 and Pcyox1l expression in DVT mice by RT-qPCR and Western blot, and revealed that Pcyox1l was significantly upregulated in the femoral venous blood of DVT mice (Fig. 3D, E). Immunohistochemistry results (Fig. 3F, G) showed that the positive signal of brown or dark brown particles indicated the expression of Pcyox1l protein. Optical density analysis found that the integrated optical density (IOD) of Pcyox1l in DVT mice was significantly higher than that in sham-operated mice.

Finally, we obtained miRNAs that could bind to Crnde and Pcyox1l respectively through RNAInter database, and then miRNAs to Pcyox1l through mirWalk database. Taking the intersection of the three datasets, we finally selected the key gene miR-181a-5p (Fig. 3H, Table S3). Finally, the ceRNA regulatory network of Crnde/miR-181a-5p/Pcyox1l involved in DVT is constructed.

### Overexpression of miR-181a-5p attenuated vascular inflammatory injury, thereby curtailing thrombus formation

**Fig. 4:**
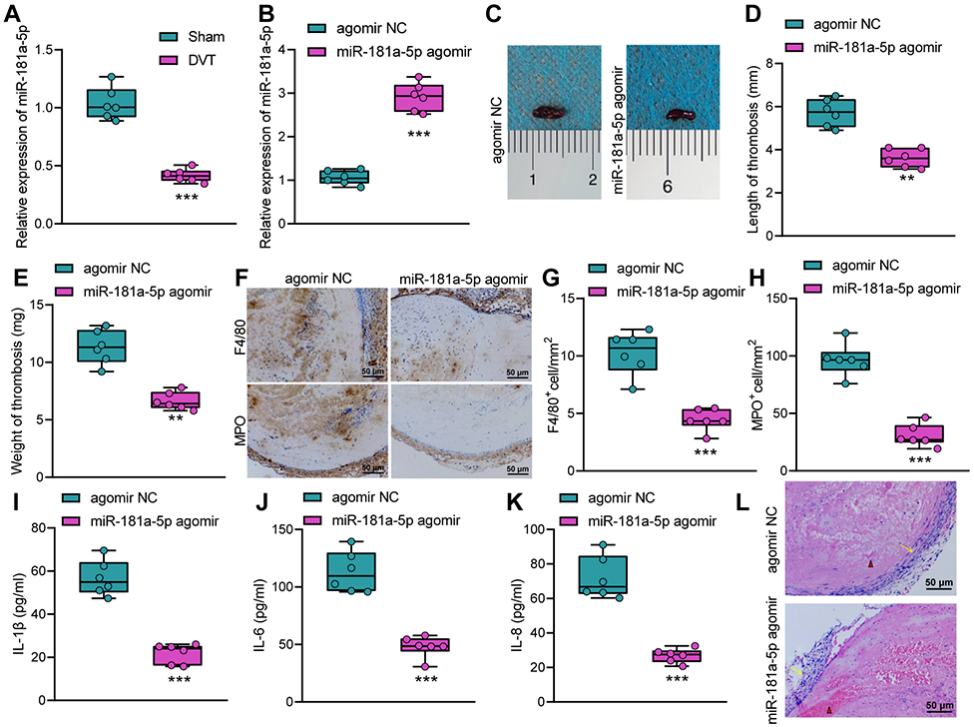
Overexpression of miR-181a-5p attenuates vascular inflammatory injury, thereby curtailing DVT. A, RT-qPCR detection of miR-181a-5p expression in the inferior vena cava tissues of the sham-operated mice and DVT mice. B, RT-qPCR detection of miR-181a-5p expression in the inferior vena cava tissues of the miR-181a-5p agomir-treated DVT mice. C, Representative image of thrombosis in inferior vena cava of the miR-181a-5p agomir-treated DVT mice. D & E, Measurement results of length (D) and weight (E) of thrombus in the miR-181a-5p agomir-treated DVT mice. F, Representative micrographs of thrombus (F4/80) and myeloperoxidase staining for the miR-181a-5p agomir-treated DVT mice. G, Quantification of F4/80 positive cells per unit area in the inferior vena cava of the miR-181a-5p agomir-treated DVT mice. H, Quantification of myeloperoxidase staining-positive cells per unit area in the inferior vena cava of the miR-181a-5p agomir-treated DVT mice. I-K, The levels of inflammatory factors IL-1β (I), IL-1, IL-6 (J) and IL-8 (K) in the femoral venous blood of the miR-181a-5p agomir-treated DVT mice. L, The vascular inflammatory injury and platelet bundles in the miR-181a-5p agomir-treated DVT mice. Red triangles indicate clustered red blood cells, and yellow arrows indicate the blood vessel wall. ** *p* < 0.01, *** *p* < 0.001 vs. the sham group or agomir NC group. n = 6

We further explored the role of miR-181a-5p in DVT. RT-qPCR results (Fig. 4A) showed that miR-181a-5p expression was notably downregulated in inferior vena cava tissue of DVT mice compared with that in the sham-operated mice.

Next, we injected either the lentiviral vector harboring agomir NC or miR-181a-5p agomir in mice. Based on RT-qPCR results, miR-181a-5p was upregulated in the DVT mice after treatment with miR-181a-5p agomir (Fig. 4B). Moreover, the weight and the length of the thrombus in the inferior vena cava of the DVT mice were notably reduced after treatment with miR-181a-5p agomir (Fig. 4C-E).

Immunohistochemical staining results displayed that inflammatory cells, especially macrophages and neutrophils, were decreased in the thrombus in the inferior vena cava of the miR-181a-5p agomir-treated DVT mice (Fig. 4F-H). ELISA found that the levels of inflammatory factors IL-1β, IL-6 and IL-8 decreased significantly in the serum of miR-181a-5p agomir-treated DVT mice (Fig. 4I-K). The HE staining results (Fig. 4L) showed that miR-181a-5p agomir-treated DVT mice had lighter staining of the thrombosis, diminished platelet bundles, and decreased inflammatory cell infiltration. Therefore, overexpressed miR-181a-5p attenuated vascular inflammatory injury to curtail DVT.

### Crnde competitively bound to miR-181a-5p to release Pcyox1l expression

**Fig. 5:**
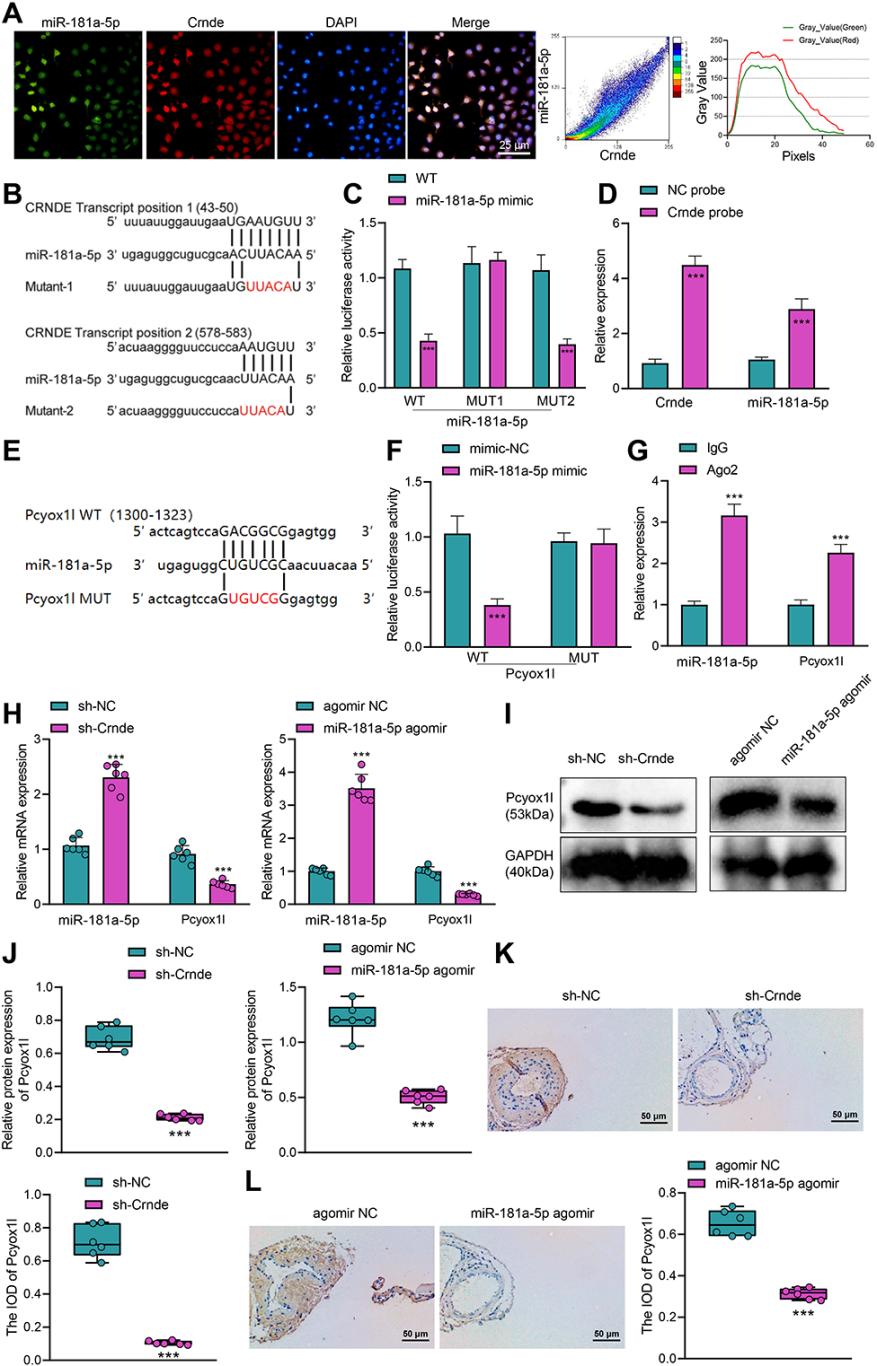
Validation of the targeting relationship between Crnde and miR-181a-5p and that between miR-181a-5p and Pcyox1l. A, RNA-FISH assay for detection of the co-localization of Crnde and miR-181a-5p in the nucleus and quantitative statistical analysis results. B, The binding site and mutation site of Crnde and miR-181a-5p. C, Dual luciferase reporter gene assay to verify the binding of Crnde to miR-181a-5p. D, The direct binding between Crnde and miR-181a-5p detected by RNA pull-down assay. E, The binding site and mutation site of Pcyox1l and miR-181a-5p. F, Dual luciferase reporter gene assay to verify the binding of Pcyox1l mRNA to miR-181a-5p. G, The interaction relationship between Pcyox1l mRNA to miR-181a-5p as detected by RIP assay. H, Pcyox1l and miR-181a-5p expression in the DVT mice in response to Crnde knockdown or miR-181a-5p agomir measured by RT-qPCR. I & J, Images (I) and quantitation (J) of the Pcyox1l expression in the DVT mice in response to Crnde knockdown or miR-181a-5p agomir determined by Western blot. K & L, Images (K) and quantitation (L) of Pcyox1l protein expression in the DVT mice in response to Crnde knockdown or miR-181a-5p agomir determined by immunohistochemistry. *** *p* < 0.001 vs. the mimic-NC, NC probe, sh-NC or agomir NC group. n = 6. All cell experiments were independently repeated three times

Next, we validated the targeting relationship between Crnde and miR-181a-5p. First, we co-localized the location of Crnde and miR-181a-5p in the cytoplasm by RNA-FISH assay (Fig. 5A). Afterwards, we used the LncBase Predicted v.2 of the DIANA tool to obtain the binding targets of Crnde and miR-181a-5p, with two putative miR-181a-5p binding sites in the regions 43–35 and 578–583 of Crnde (Fig. 5B). We constructed Crnde luciferase plasmids containing the potential miR-181a-5p binding site and its mutants of each site, and co-transfected these plasmids with miR-181a-5p into vascular endothelial cells, followed by dual luciferase reporter gene assays. As shown in Fig. 5C, miR-181a-5p reduced the activity of both WT and MUT2 Crnde luciferase, but it could not affect the MUT1 Crnde luciferase activity, indicating that miR-181a-5p could bind to Crnde (43–50). We used the Crnde-specific probes for RNA precipitation in the cells, and the results of the RNA pull-down assay (Fig. 5D) showed that the miR-181a-5p and Crnde were significantly enriched. The aforementioned results suggested that Crnde could targetedly bind to miR-181a-5p.

We further validated the targeting relationship between Pcyox1l mRNA and miR-181a-5p. We obtained the binding targets for Pcyox1l mRNA and miR-181a-5p through the mirWalk database (Fig. 5E). Dual luciferase reporter gene assay results (Fig. 5F) demonstrated that miR-181a-5p reduced the WT Pcyox1l luciferase activity, but it failed to affect the MUT Pcyox1l luciferase activity. Meanwhile, the RIP assay results (Fig. 5G) displayed that Ago2 could simultaneously enrich miR-181a-5p and Pcyox1l mRNA. These results indicated that miR-181a-5p could target and bind to Pcyox1l mRNA.

Furthermore, Crnde silencing or treatment with miR-181a-5p agomir resulted in enhanced miR-181a-5p expression and decreased Pcyox1l expression in the DVT mice (Fig. 5H-J). From the immunohistochemistry results (Fig. 5K, L), the positive signal of brown or dark brown particles indicated the expression of Pcyox1l protein. Furthermore, the IOD of Pcyox1l was markedly diminished in the DVT mice with Crnde knockdown or miR-181a-5p overexpression. The above results demonstrated that miR-181a-5p could target Pcyox1l mRNA and inhibit its expression.

### Overexpression of Pcyox1l reversed the inhibitory effect of Crnde silencing on DVT in mice

**Fig. 6:**
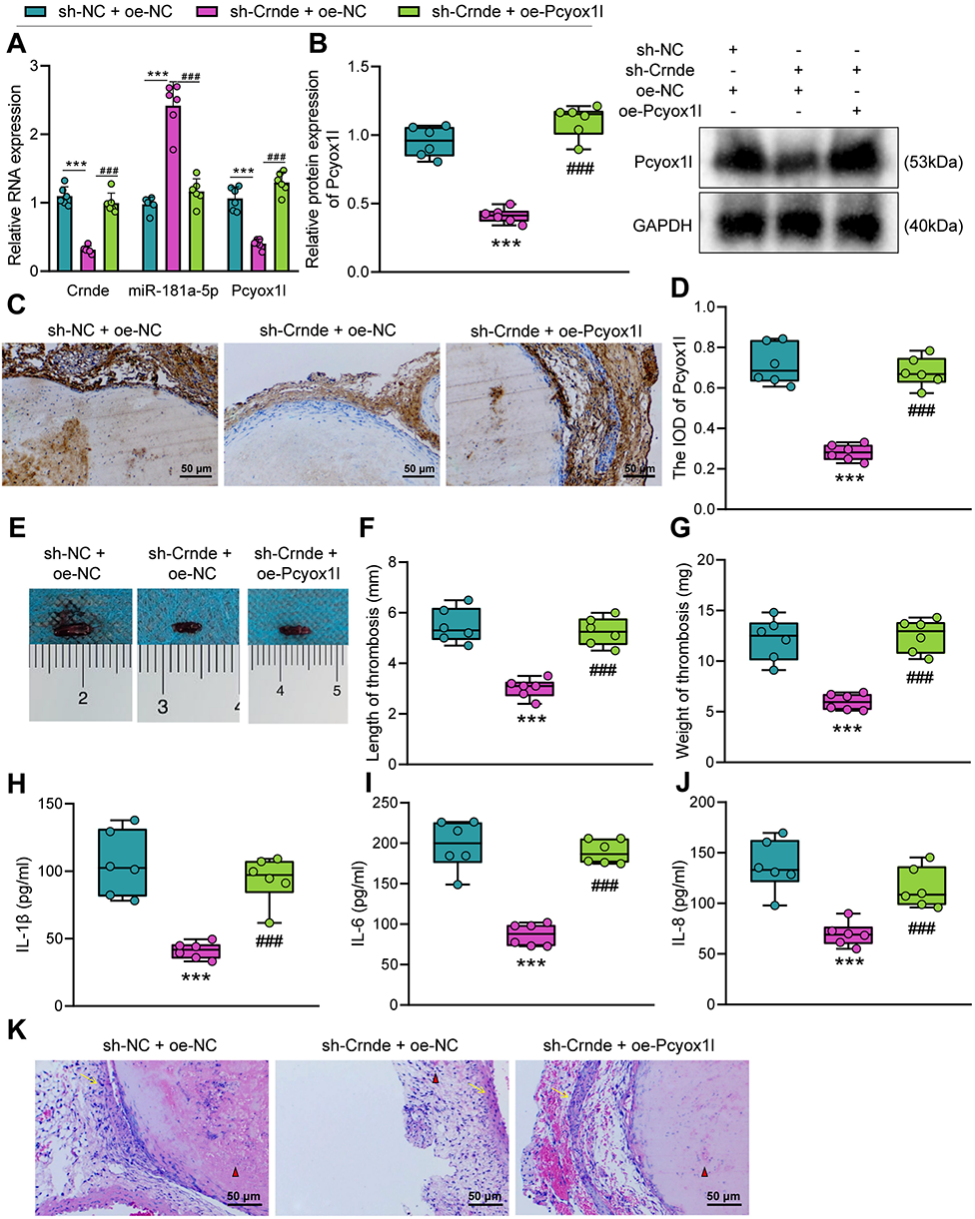
Overexpression of Pcyox1l reverses the inhibitory effect of Crnde silencing on DVT in mice. A, The expression of Crnde, miR-181a-5p and Pcyox1l in DVT mice treated with sh-Crnde alone or combined with oe-Pcyox1l determined by RT-qPCR. B, Western blot for the Pcyox1l protein expression in DVT mice treated with sh-Crnde alone or combined with oe-Pcyox1l. C & D, Images (C) and quantitation (D) of Pcyox1l protein expression in the inferior vena cava tissue of the DVT mice treated with sh-Crnde alone or combined with oe-Pcyox1l determined by immunohistochemistry. E, Representative image of thrombosis in inferior vena cava of the DVT mice in response to Crnde knockdown alone or combined with Pcyox1l overexpression. F & G, Measurement results of thrombus length (F) and thrombus weight (G) of the DVT mice in response to Crnde knockdown alone or combined with Pcyox1l overexpression. H-J, The levels of inflammatory factors IL-1β (H), IL-6 (I) and IL-1 and IL-8 (J) in the femoral venous blood of the DVT mice in response to Crnde knockdown alone or combined with Pcyox1l overexpression measured by ELISA. K, HE staining showing vascular inflammatory injury and platelet bundles in the DVT mice in response to Crnde knockdown alone or combined with Pcyox1l overexpression. Red triangles indicate the clustered red blood cells, and yellow arrows indicate the blood vessel wall. *** *p* < 0.001 vs. the sh-NC + oe-NC group. ### *p* < 0.001 vs. the sh-Crnde + oe-NC group. n = 6

To further validate the role of Crnde in DVT by regulating the miR-181a-5p/Pcyox1l axis, we injected lentivirus expressing sh-NC + oe-NC, sh-Crnde + oe-NC, and sh-Crnde + oe-Pcyox1l into DVT mice. The Crnde and Pcyox1l expression was elevated while miR-181a-5p expression was decreased in the presence of sh-Crnde + oe-Pcyox1l (Fig. 6A, B). Immunohistochemistry (Fig. 6C, D) showed that positive signals of brown or dark brown particles were indicative of Pcyox1l protein expression, mainly in the vascular endothelium and smooth muscle, accompanied by inflammatory cell infiltration in the blood clot and around the blood vessel wall. By immunohistochemistry analysis, the IOD of Pcyox1l was markedly diminished in response to combined Crnde knockdown and Pcyox1l overexpression.

As illustrated in Fig. 6E-G, the thrombus length and weight were increased by combined Crnde knockdown and Pcyox1l overexpression. The results of ELISA (Fig. 6H-J) showed increased serum levels of IL-1β, IL-6, and IL-8 upon combined Crnde knockdown and Pcyox1l overexpression. In addition, the HE staining results (Fig. 6K) found that the histopathological changes of femoral vein tissue were increased in response to combined Crnde knockdown and Pcyox1l overexpression. Collectively, Crnde could promote thrombosis through the Crnde/miR-181a-5p/Pcyox1l axis.

**Fig. 7:**
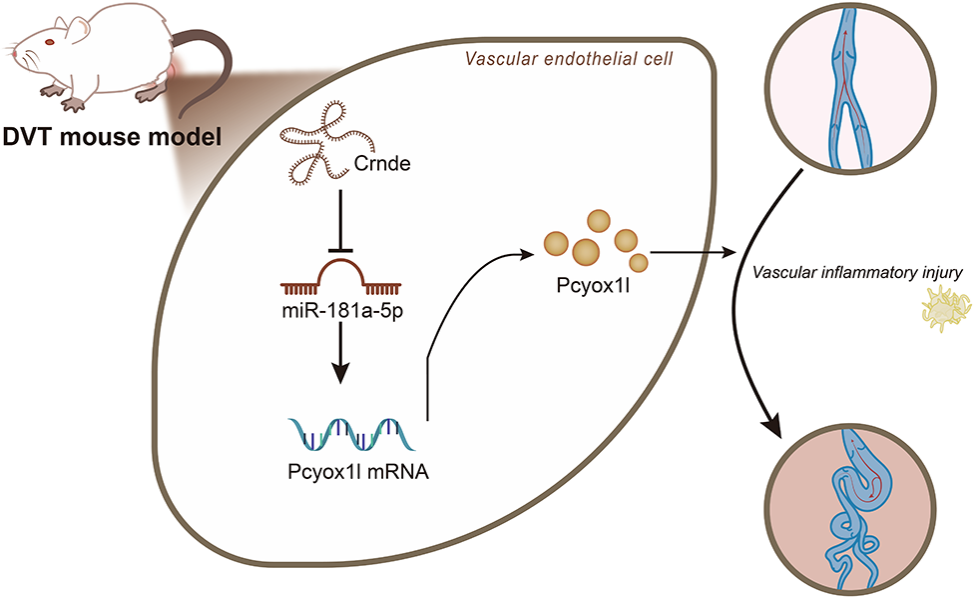
Schematic representation of the molecular mechanism of the Crnde/miR-181a-5p/Pcyox1l ceRNA regulatory network in DVT. LncRNA Crnde upregulates Pcyox1l expression through competitive binding to miR-181a-5p, thus aggravating vascular inflammatory injury and eventually leading to DVT.

## Discussion

DVT, a coagulation disorder, is related to inflammation and causes morbidity and mortality [29]. Our study intended to reveal the possible molecular mechanism of the Crnde/miR-181a-5p/Pcyox1l ceRNA regulatory network in DVT, which found that Crnde could promote thrombus formation through the /miR-181a-5p/Pcyox1l axis.

Initially, our high-throughput transcriptome sequencing analysis identified that Crnde was involved in DVT. We further validated that silencing of Crnde ameliorated vascular inflammatory injury, thereby curtailing DVT. Accumulating evidence has indicated the implication of lncRNAs in DVT. For instance, repression of lncRNA 1123 was unveiled to constrain lower extremity DVT by regulating miR-125a-3p to target interleukin 1 receptor type 1 [19]. Additionally, lncRNA MALAT1 affected the vascular endothelial cell physiology in DVT via the miR-383-5p/BCL2L11 axis [30]. It should be noted that the role of Crnde in DVT has been rarely reported. Nevertheless, the association of Crnde with inflammation has been increasingly revealed. Knockdown of Crnde contributed to amelioration of inflammation injury induced by LPS in WI-38 cells by mediating FOXM1 [31]. Crnde induced inflammation in HK-2 cells via activation of the TLR4/NF-κB axis [32]. Interestingly, Crnde was found to be upregulated in injured rat carotid artery as well as vascular smooth muscle cells [9].

Further bioinformatics analysis predicted that the Crnde/miR-181a-5p/Pcyox1l ceRNA regulatory network might participate in DVT. In addition, overexpression of miR-181a-5p was revealed to attenuate vascular inflammatory injury, thereby curtailing DVT. miR-181 was previously suggested to be a promising therapeutic target for curtailing thrombosis [14]. Vascular miR-181b could control tissue factor-dependent thrombogenicity as well as inflammation in type 2 diabetes [33]. Besides, miR-181a-5p modulated the angiogenesis of human umbilical vein endothelial cells by targeting PDGFRA [34]. Moreover, miR-181a-5p alleviated inflammatory response in pulmonary arterial hypertension induced by monocrotaline, which was achieved by targeting endocan [16]. These reports can support our finding regarding the alleviatory role of miR-181a-5p in vascular inflammatory injury in DVT.

Mechanistically, we found in this study that Crnde could upregulate Pcyox1l expression by competitively binding to miR-181a-5p. The loss of Pcyox1 could cause platelet hypo-reactivity or impairment of arterial thrombosis, and Pcyox1 might be developed as an antithrombotic drug [12]. Notably, deficiency in Pcyox1 in an ApoE mouse model inhibited atheroprogression, which was achieved partly by reducing inflammation and regulating platelet adhesion [35]. Pcyox1l is rarely researched, though a previous study investigated the potential prognostic potential of the lncRNA transcript lnc-Pcyox1l in clear cell renal cell carcinoma but failed to find its differential expression in the malignancy [36]. LncRNAs can modulate biological functions at epigenetic, transcriptional or post-transcriptional levels and miRs can affect physiological and pathological processes by mediating target mRNA translation or degradation [37]. LncRNA transcripts serve as ceRNAs or natural miR sponges and competitively bind to shared miRs to co-regulate each other [38]. To our knowledge, previous studies have indicated the interaction between Crnde and miR-181a-5p under different situations. Crnde could sponge miR-181a-5p, contributing to aggravation of sepsis-related inflammation [13]. Moreover, Crnde downregulated the expression of miR-181a-5p to facilitate the proliferation and chemoresistance of colorectal cancer cells, with the involvement of Wnt/β-catenin signaling [39]. It is worthy to note that there is scarcity of reports regarding the interaction between miR-181a-5p and Pcyox1l. In our study, the database-based bioinformatics analysis combined with dual luciferase reporter gene and RIP assays confirmed the targeting relationship between miR-181a-5p and Pcyox1l.

## Conclusion

Taken together, the present study provides evidence suggesting that Crnde competitively bound to miR-181a-5p to upregulate Pcyox1l expression, thereby aggravating vascular inflammatory injury and the DVT (Fig. 7). Thess findings are likely to provide novel molecular targets for the diagnosis and treatment of DVT. However, the specific mechanism of Pcyox1l in DVT still needs further validation.

## Electronic supplementary material

Below is the link to the electronic supplementary material.

Figure S1 Identification of primary endothelial cells. A, The morphology of primary endothelial cells observed under alight microscope. B, The positive expression of endothelial marker proteins CD31 and VE-Cadherin in primary endothelial cells observed under a fluorescence microscope. The green color indicates CD31 or VE-Cadherin, and the blue color indicates DAPI. The cell experiments were independently repeated three times

Supplementary Material 2

Supplementary Material 3

Supplementary Material 4

## Data Availability

The data underlying this article will be shared on reasonable request to the corresponding author.
