# Supplementary material for: Long non-coding RNA crnde promotes deep vein thrombosis by sequestering miR-181a-5p away from thrombogenic Pcyox1l
Source: Thromb J. 2023 Apr 19;21:44. doi: 10.1186/s12959-023-00480-9 (PMC10116699; doi:10.1186/s12959-023-00480-9)
Supplement: Supplementary file 2 — Supplementary Material 2 [file 12959_2023_480_MOESM2_ESM.docx]

**Table S1** shRNA sequences

| shRNA | Sequence |
| --- | --- |
| sh-Crnde | 5'-CACCGGAAGGAGGAGATTCTGAAGATTCAAGAGATCTTCAGAATCTCCTCCTTCCTTTTTG-3' |
| sh-NC | 5'-CACCGTTCTCCGAACGTGTCACGTCAAGAGATTACGTGACACGTTCGGAGAATTTTTTG-3' |

Note: sh-, shRNA short hairpin RNA; NC, negative control.
