# Supplementary material for: Long non-coding RNA crnde promotes deep vein thrombosis by sequestering miR-181a-5p away from thrombogenic Pcyox1l
Source: Thromb J. 2023 Apr 19;21:44. doi: 10.1186/s12959-023-00480-9 (PMC10116699; doi:10.1186/s12959-023-00480-9)
Supplement: Supplementary file 3 — Supplementary Material 3 [file 12959_2023_480_MOESM3_ESM.docx]

**Table S2** Primer sequences for RT-qPCR

| Gene | Sequence |
| --- | --- |
| Srrm4os | Forward: 5′-AGGTGGTTCATGCAAATGGC-3′ |
|  | Reverse: 5′-GCTCCAACATGGCTGCAATC-3′ |
| Six3os1 | Forward: 5′-AGCAGGGAACTCTCGGTTCT-3′ |
|  | Reverse: 5′-CGTCACTCCAAGCGGACAT-3′ |
| Hoxa11os | Forward: 5′-ATCTGGACCCGAGACGTAGT-3′ |
|  | Reverse: 5′-TGACGATCTGTTGCTTCCCC-3′ |
| Emx2os | Forward: 5′-GGGCAGGTATAGTGCCTCTG-3′ |
|  | Reverse: 5′-TGAACAATCCACAGGCCCAG-3′ |
| Hottip | Forward: 5′-AGACGAAGTACGGTTCCAGG-3′ |
|  | Reverse: 5′-GGATGGGGGAATTGAGAGCG-3′ |
| Snhg17 | Forward: 5′-GGCACCAAGCATCCTACCAA-3′ |
|  | Reverse: 5′-AATCTGTCGACCCAAGGAGC-3′ |
| Akt2 | Forward: 5′-ACCCCCAGACCGATATGACA-3′ |
|  | Reverse: 5′-CTCGGATGCTGGCTGAGTAG-3′ |
| Tgfb1 | Forward: 5′-ACTGGAGTTGTACGGCAGTG-3′ |
|  | Reverse: 5′-GGGGCTGATCCCGTTGATTT-3′ |
| Med25 | Forward: 5′-TCTCTGCAGGCAGGCACT-3′ |
|  | Reverse: 5′-GAGGACTCCACTCCAGGCTA-3′ |
| Itgb4 | Forward: 5′-GGAGACCTGGAAGGAGTTGC-3′ |
|  | Reverse: 5′-ACTCCTGTCCGTTTCATCGAG-3′ |
| Asb6 | Forward: 5′-CTTCCTGCTCGGTGAGACTG-3′ |
|  | Reverse: 5′-AAGAGCTGCGTGACTTGGAA-3′ |
| Xlr | Forward: 5′-TGTAGTCAGAGCCAGACCCT-3′ |
|  | Reverse: 5′-TGTTGGTCTCCAAGTTCATCAGA-3′ |
| Crnde | Forward: 5′-CAGCTACTGTTCCCATCGAGA-3′ |
|  | Reverse: 5′-AGAGCTGCACTGATAGGTTGG-3′ |
| Pcyox1l | Forward: 5′-CAAGCTGCTGGGACTGAGAC-3′ |
|  | Reverse: 5′-CAGCAGGTACCAGTCGGTTT-3′ |
| miR-181a-5p | Forward: 5′-AACATTCAACGCTGTCGGTGAGT-3′ |
|  | Reverse: Universal reverse primer |
| U6 | Forward: 5′-CTCGCTTCGGCAGCACA-3′ |
|  | Reverse: Universal reverse primer |
| GAPDH | Forward: 5′-GGAGAGTGTTTCCTCGTCCC-3′ |
|  | Reverse: 5′-ATGAAGGGGTCGTTGATGGC-3′ |

Note: RT-qPCR, reverse transcription-quantitative polymerase chain reaction.
